# Carbon-Related Quantum Emitter in Hexagonal Boron Nitride with Homogeneous Energy and 3-Fold Polarization

**DOI:** 10.1021/acs.nanolett.3c03628

**Published:** 2024-01-19

**Authors:** Ding Zhong, Shiyuan Gao, Max Saccone, Julia R. Greer, Marco Bernardi, Stevan Nadj-Perge, Andrei Faraon

**Affiliations:** † Thomas J. Watson, Sr., Laboratory of Applied Physics, California Institute of Technology, Pasadena, California 91125, United States; ‡ Kavli Nanoscience Institute, California Institute of Technology, Pasadena, California 91125, United States; § Institute for Quantum Information and Matter, California Institute of Technology, Pasadena, California 91125, United States; ∥ Department of Applied Physics and Material Science, California Institute of Technology, Pasadena, California 91125, United States; ⊥ Division of Chemistry and Chemical Engineering, California Institute of Technology, Pasadena, California 91125, United States; # Division of Engineering and Applied Science, California Institute of Technology, Pasadena, California 91125, United States

**Keywords:** Single-photon emitters, Hexagonal boron nitride, Carbon defect, Three-level systems

## Abstract

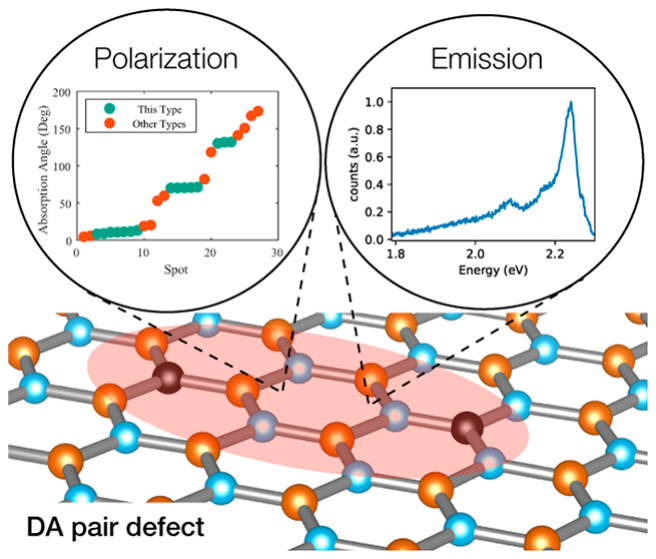

Most hexagonal boron nitride (hBN) single-photon emitters (SPEs) studied to date suffer from variable emission energy and unpredictable polarization, two crucial obstacles to their application in quantum technologies. Here, we report an SPE in hBN with an energy of 2.2444 ± 0.0013 eV created via carbon implantation that exhibits a small inhomogeneity of the emission energy. Polarization-resolved measurements reveal aligned absorption and emission dipole orientations with a 3-fold distribution, which follows the crystal symmetry. Photoluminescence excitation (PLE) spectroscopy results show the predictability of polarization is associated with a reproducible PLE band, in contrast with the non-reproducible bands found in previous hBN SPE species. Photon correlation measurements are consistent with a three-level model with weak coupling to a shelving state. Our ab initio excited-state calculations shed light on the atomic origin of this SPE defect, which consists of a pair of substitutional carbon atoms located at boron and nitrogen sites separated by a hexagonal unit cell.

Since their discovery,^1−4^ single-photon emitters (SPEs) in van der Waals materials such as hexagonal boron nitride (hBN) have seen a rapid development.^5−19^ This platform exhibits a high photon extraction rate and enables on-chip engineering based on electric fields,^20^ magnetic fields,^21^ doping,^22^ and strain,^23−25^ with versatile control of the SPE properties. These SPEs are stable when the material is transferred, enabling integration with photonic devices^26−28^ or van der Waals heterostructures^29^ for complex systems. Progress has also been made to suppress their spectral diffusion^30^ and blinking.^31^ However, a primary challenge limiting the application of these SPEs is large inhomogeneity, which is believed to originate from possibly two main sources: environmental fluctuations, such as strain and electrostatic noise, or the presence of multiple species within the emitters.^32^ In terms of energy, their emission energy ranges seemingly randomly from 1.66 to 2.20 eV.^1,33^ Additionally, their optical dipole orientations show significant randomness,^34^ further adding to their unpredictability. In fact, no clear correlation has been identified between their dipole orientations and the crystallographic axes of hBN.^35,36^ These disadvantages not only add complexities to device fabrication, often necessitating preselection based on desired wavelength and polarization, but also pose challenges to achieving coherence between emitters. Such coherence is essential for applications such as quantum computing,^37^ quantum networking^38^ and quantum metrology.^39^

Here, we report the identification of a new type of quantum emitters in carbon-implanted, layered hBN. Using photoluminescence, we first identify a cluster of emitters centered around 2.24 eV. These emitters also exhibit a strong correlation between optical dipole orientation and crystal directions with 3-fold symmetry in hBN. Photoluminescence excitation (PLE) spectra for these emitters exhibit consistent resonances, in contrast to emitters reported in previous studies, where variations were observed.^1,6^ As for quantum characteristics, autocorrelation measurements show single-photon statistics that are consistent with a three-level system with very weak coupling to a shelving state, indicating a high single-photon generation efficiency. Low-temperature measurements show a very narrow energy distribution (2.444 ± 0.0013 eV), a substantial reduction in inhomogeneity compared to previous reported types. Ab initio calculations indicate that a pair of substitutional carbon atoms separated by a hexagonal unit cell are the most probable microscopic origin of the emitter.

## Results and Discussion

**Figure 1:**
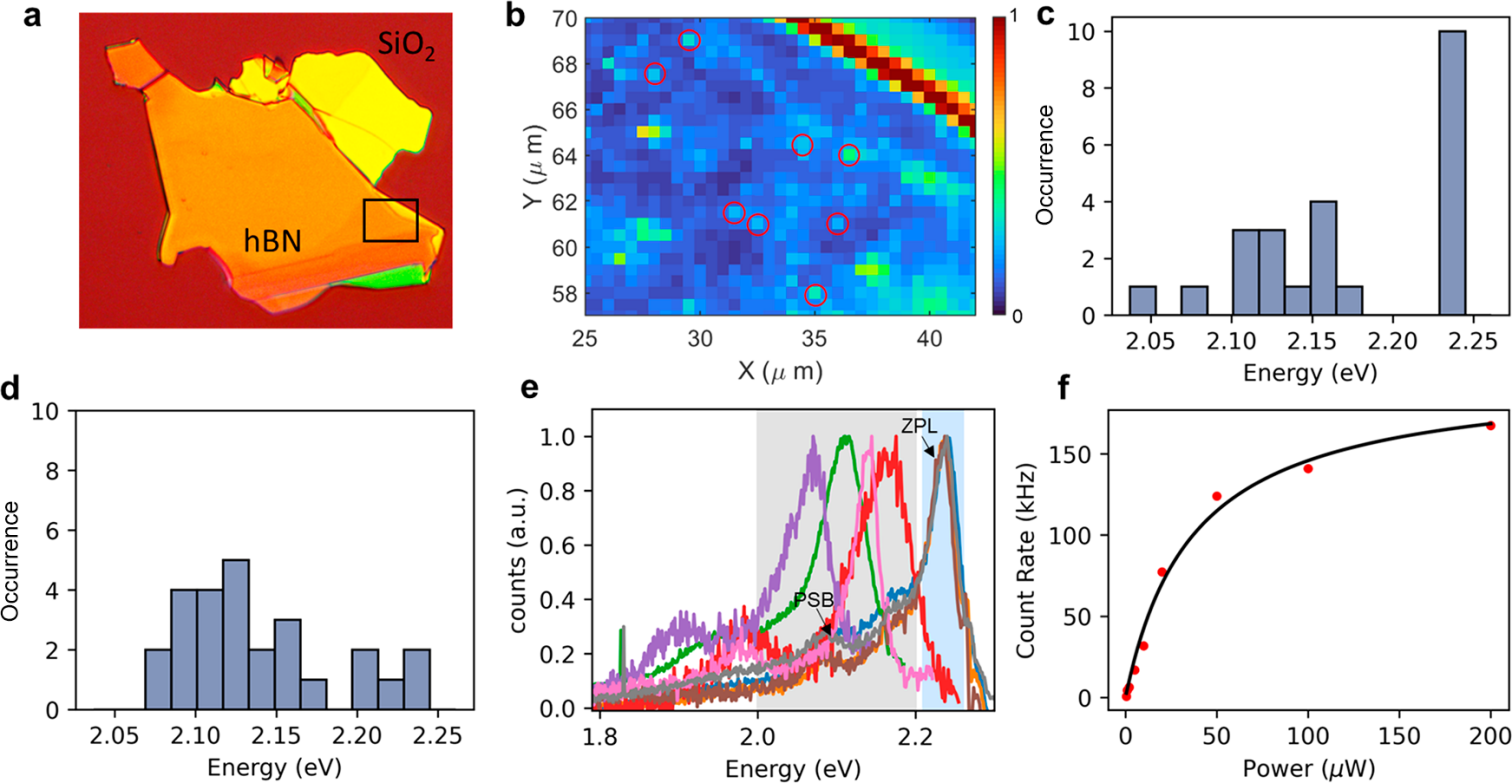
Photoluminescence characterization of the emitters. (a) Optical image of the measured hBN flake. The image has a size of 100 μm by 118 μm. (b) Photoluminescence map of the flake in the region marked with a rectangle. The red circles mark the locations of the emitters. (c) Energy distribution of multiple emitters from the flake. (d) Energy distribution of multiple emitters from a ^16^O^+^-implanted flake. (e) Representative spectra of four type I emitters (blue shaded) and four type II emitters (gray shaded). (f) Count rate as a function of laser power. The black curve is a fit to the data.

The emitters we studied were identified in multilayer hBN implanted with carbon 12+ and annealed (see Methods for details). Using 532 nm continuous-wave laser excitation, we performed raster scans over the hBN flakes at room temperature to identify the locations of the emitters. Figure 1a shows an optical image of a representative hBN flake. Figure 1b shows a portion of the raster scan map on the flake with its color representing the average intensity between [1.8, 2.25] eV for each pixel, where several representative local intensity hotspots corresponding to the emitter locations are circled. The peak energy distribution of 24 emitters from the flake is plotted in Figure 1c. While many of them are distributed between 2.18 eV (568 nm) and 2.00 eV (620 nm), a large fraction is centered around 2.24 eV (553 nm). This concentration of emitters with energy around 2.24 eV is absent in a control sample where ^16^O^+^ was implanted with the same parameters, as shown in Figure 1d. (for more details, see the Supporting Information). We attribute this class of homogeneous emitters at 2.24 eV to a new type of defect color center and refer to them as “type I”. We attribute the other class of emitters with large inhomogeneity in emission energy to previously reported quantum emitter type(s) in hBN^1,5,40^ and refer to them as “type II” in this work.

Figure 1e displays the normalized spectra from representative emitters of both types. The type I emitters display a more homogeneous peak shape than the type II emitters in terms of both peak energy and peak width. Both types of emitters exhibit an energy gap of ∼165 meV between the zero-phonon line (ZPL) and the phonon sideband (PSB). This is expected, as both emitters couple to the same optical phonons in hBN, and based on calculations,^41^ the density of states (DOS) of hBN phonons has a maximum at 165 meV. Figure 1f shows the saturation curve of a type I emitter. We fit the experimental data to the function *I*(*P*) = *I*_∞_ × *P*/(*P* + *P*_S_), where *I*_∞_ is the count rate at infinite excitation power and *P*_S_ is the excitation power at saturation. The fitting gives values of *P*_S_ = 37 μW and *I*_∞_ = 200 kHz.

**Figure 2:**
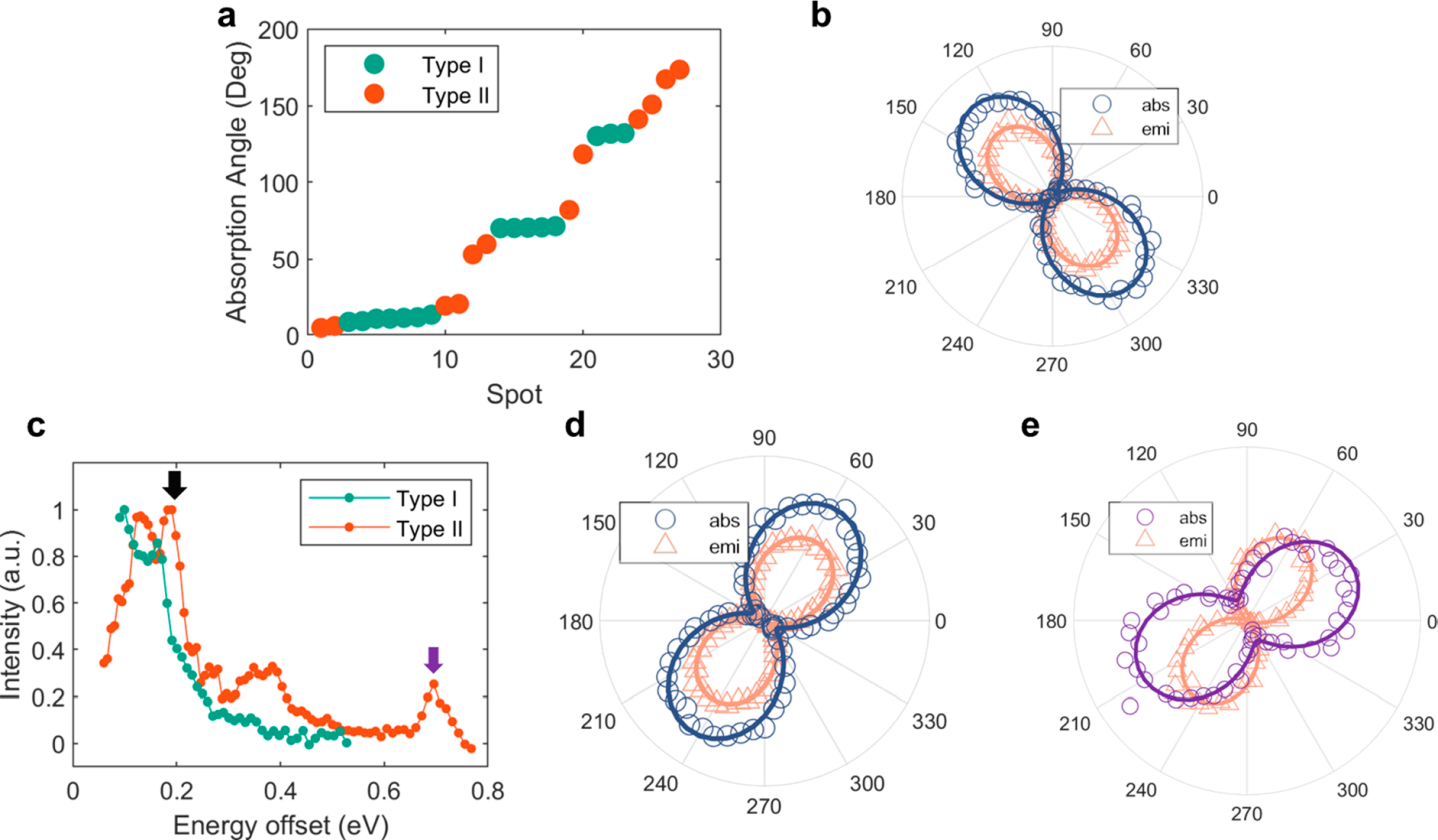
Polarization and photoluminescence excitation characterization. (a) The absorption polarization of 27 emitters with their types color-coded, excited by a 2.33 eV laser. (b) Typical ZPL intensity as a function of excitation/detection polarization angle for a type I emitter. The axis for the maximum intensity for excitation/detection polarization indicates the intrinsic absorption/emission dipole orientation of the emitter. (c) Photoluminescence excitation of a type I and type II emitter plotted against the energy offset from their respective emission energy (2.00 eV for type II and 2.24 eV for type I). (d) The absorption polarization and emission polarization when the type II emitter is excited at 2.19 eV (0.19 eV offset), as indicated by the black arrow in (c). (e) The absorption and emission dipole orientations for the type II emitter at a 2.69 eV excitation energy (0.69 eV offset), as indicated by the purple arrow in (c).

Measurements under linear polarization excitation on these two types of emitters revealed distinct properties. By tracking the detected ZPL intensity while controlling the polarization of the excitation laser or measuring the polarization of the detected PL signal, we characterized both the absorption dipole orientation and the emission dipole orientation (experimental details are given in the Methods section). In Figure 2a, for an ensemble of emitters on a single flake, we show the absorption dipole orientations of multiple emitters with the emitter type color-coded. For type I emitters, the absorption dipole orientation only takes three discrete values, 11°, 71°, and 131° (±1°), which are 60° apart (the angular offset is arbitrary). In contrast, for type II emitters, we found that the dipole orientations were distributed across the entire range without an obvious pattern, which is in agreement with previously reported SPEs in hBN.^35^ The 3-fold distribution for the type I SPEs is consistent with the crystal symmetry of the hBN host lattice, suggesting a symmetric atomic structure for the type I SPEs. We also found that the absorption dipole orientation was always aligned to the emission dipole orientation in type I emitters (Figure 2b), while for type II emitters, this behavior was observed only occasionally.^34,35^ In order to show that this polarization feature does not originate from energy preselection of emitters, in the Supporting Information, we present the results of 10 energy-preselected type II emitters. This data reveals no discernible pattern in the absorption or emission dipole orientations. Defining the polarization degree as *p* = (*I*_max_ – *I*_min_)/(*I*_max_ + *I*_min_), we obtained absorption and emission polarization values of *p* = 96% for type I emitters, which is strong evidence for a linearly polarized dipole-like emitter.

PLE spectroscopy was performed, and it was found that these consistently aligned absorption–emission dipole orientations for the type I emitters were associated with their repeatable excitation resonances. In PLE spectroscopy, the PL can be detected if the system is excited by light with the proper wavelength and relaxes onto the excited state corresponding to the ZPL emission. We repeatedly obtained identical PLE spectra for each individual type I emitter, with one example shown in Figure 2c in cyan (more examples are shown in the Supporting Information). In the spectrum from 2.33 (0.09 eV offset) to 2.77 eV (0.53 eV offset), we observed only one resonant peak close to the emission energy, and the PL intensity dropped as the excitation energy increased. In addition, the absorption dipole orientation over the entire excitation resonance peak was found to always align with the emission dipole orientation of the PL.

In contrast, the PLE spectra for the type II emitters varied between individual emitters. In Figure 2c, we also show the PLE spectrum (orange plot) from a randomly selected type II emitter (emission energy: 2.00 eV; more examples are shown in the Supporting Information). The spectrum consists of a continuum extending from (close to) the emission energy to around a 0.48 eV offset energy plus a separate peak at 0.69 eV. We found that the absorption dipole orientation within the continuum was identical to the emission dipole orientation (Figure 2d). We measured a different dipole orientation for the peak at 0.69 eV, although the emission polarization did not change with the excitation energy, as shown in Figure 2e.

These results can be explained in the framework of the Franck–Condon principle.^42^ Within the Born–Oppenheimer approximation, where the nuclei and electronic wave functions are treated as independent, the probability amplitude for the decay process can be written as
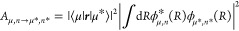
1where *R* is the nuclear coordinate and ϕ_μ,*n*_ is the combined wave function for an electronic state μ and vibronic state with *n* phonons occupied. For a type I emitter, the excitation is between (μ_0_, 0) and (μ_1_, *n*_1_), which means the emitter is first excited to a higher vibronic sublevel of the excited state, followed by fast vibrational relaxation, and then the ZPL is radiated. The identical absorption and emission dipole moments are a consequence of |⟨μ_0_|***r***|μ_1_⟩|^2^ possessing time-reversal symmetry. For a type II emitter, the continuum below 0.48 eV offset energy is similarly a transition between (μ_0_′, 0) and (μ_1_′, *n*_1_′). However, for the resonance peak at 0.69 eV offset energy, the distinct absorption dipole orientation and the clear isolation of this peak suggest that it corresponds to a higher electronic state (μ_2_′, *n*_2_′) with a different transition dipole. Following Kasha’s rule,^43^ the system still relaxes to the lower excited state (μ_1_′, 0) first before it emits a photon with energy and polarization related only to the ZPL transition, such that the emission dipole is independent of excitation energy. Previous studies^35^ on type II emitters have found that the excitation energy offset with respect to emission energy is positively correlated with misaligned absorption and emission dipole orientations. From our measurements, we tentatively argue that this is because higher electronic states are more likely to be accessed with a higher excitation energy, which have a different excitation dipole orientation.

**Figure 3:**
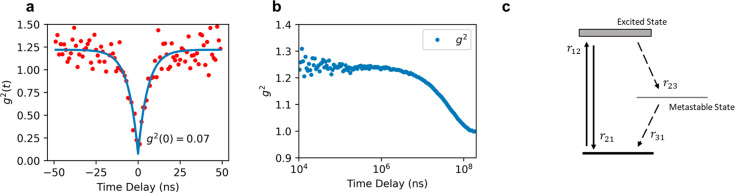
Photon autocorrelation on type I emitters. (a) Autocorrelation on a short time scale featuring antibunching. The red dots represent the experimental data, which are the number of coincidences normalized by coincidence rate at time infinity. The blue curve is the fit to the data, suggesting *g*^2^(0) = 0.07. (b) Autocorrelation on a long time scale featuring bunching. The left side of the curve is noisier due to a smaller bin size compared to the right side. (c) Schematic of a three-level model. The transition rate between states *i* and *j* is denoted by *r*_*ij*_.

Next, we investigated the type I emitters using intensity autocorrelation on a Hanbury Brown–Twiss setup. Figure 3a shows a representative background-corrected *g*^2^(τ) with *g*^2^(0) = 0.07 (*g*^2^(0) = 0.23 before the correction), which is characteristic of a single-photon emitter. The background correction formula is included in the Methods section.

A bunching feature is seen on a longer time scale of 1 × 10^8^ ns, as shown in Figure 3b. The emergence of such bunching indicates the presence of a third state. In a three-level model, which includes a ground state, an excited state, and a shelving state, when excited, the emitter predominantly relaxes back to the ground state but less frequently relaxes through the shelving state. The process is illustrated in Figure 3c, with the transition rate between states *i* and *j* denoted as *r*_*ij*_. By solving the photon dynamics assuming a 1 to 2 excitation at time zero, the autocorrelation function can be expressed as^44^
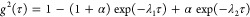
2where λ_1_ = *r*_12_ + *r*_21_ is the decay rate of the antibunching feature, λ_2_ = *r*_31_ + *r*_23_*r*_12_/(*r*_12_ + *r*_21_) is the decay rate of the bunching feature, and α = *r*_12_*r*_23_/[*r*_31_(*r*_12_ + *r*_21_)] determines the bunching amplitude. Assuming a power-dependent excitation rate *r*_12_=β*P*, we could obtain these coefficients quantitatively by conducting the experiment with different amounts of power (calculations are shown in the Supporting Information). We obtained a relaxation rate of *r*_21_= 183.3 MHz, and *r*_23_ was the order of hundreds of Hz, which is more than 5 orders of magnitude smaller than *r*_21_. The high *r*_21_/*r*_23_ ratio signifies a dominant radiative relaxation process, making it a highly efficient single-photon source.

**Figure 4:**
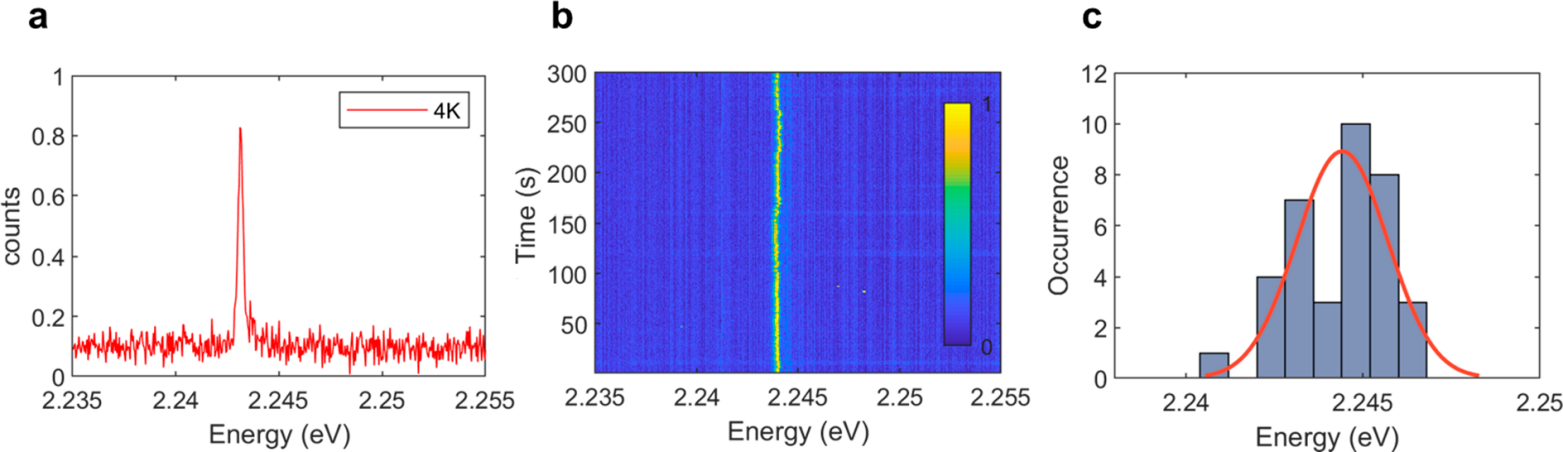
Properties of type I emitters at low temperature. (a) Low-temperature spectra of a type I emitter. (b) Time series spectra of a type I emitter for 300 s with 1 frame per second. (c) Peak energy distribution of 36 type I emitters.

We further investigated the properties of this defect at a low temperature. In Figure 4a, we show a spectrum of the type I emitter at 4 K. Besides the slightly blue-shifted emission energy, the peak width was significantly reduced due to the weaker effect of acoustic phonons at low temperatures.^47^ (Note that the line width in Figure 4a is limited by the ∼150 μeV resolution of our spectrometer, and thus, the actual line width may be even narrower.) By measuring the spectrum as a function of time, we observed the spectral diffusion of the emitter. In Figure 4b, we take 1 frame per second for 300 consecutive seconds. Minor shifts in the energy between frames indicate moderate spectral diffusion. Over the course of 300 s, we found a spectral diffusion range of ∼ 270 μeV, possibly originating from electrical transitions in nearby defects and impurities causing electrostatic fluctuations in the environment. In Figure 4c, we fitted the peak positions of 36 type I emitters, which gave a distribution of 2.2444 ± 0.0013 eV. This inhomogeneity of 0.0013 eV is 2 orders of magnitude smaller than in previously identified type II emitters.^1,33^

On the basis of these experimental results, we use first-principles calculations to look for an atomic defect with properties consistent with those of the type I emitter. This search used the following criteria:

(1) The defect contains carbon but no other external elements.
(2) To account for the distinctive absorption polarization with 60° spacing, the defect should possess *C*_2*v*_ or *C*_*s*_ symmetry with a mirror plane along the zigzag or armchair directions.
(3) The first excited state has a large in-plane transition dipole with a ZPL energy of ∼2.24 eV.
(4) No optically active higher excited state is present within 0.5 eV of the first excited state, to account for the lack of resonant peaks in the PLE spectrum.

To identify such a defect, we carried out first-principles GW plus Bethe–Salpeter equation (GW-BSE) calculations on defect-containing supercells of hBN using density functional theory (DFT) as the starting point (see Methods). Various candidate structures that met criteria (1) and (2) were initially considered. We used the symbol X_Y_ to denote a lattice site normally occupied by element Y (= N, B) in the pristine lattice substituted by element X (= B, N, C, V, with V standing for vacancy). Using this notation, candidate structures included double-site defects C_N_V_B_, C_N_N_B_, B_N_C_B_, V_N_C_B_, and C_N_C_B_; symmetric triple-site defects C_N_V_B_C_N,_ C_B_V_N_C_B_, C_N_C_B_C_N_ (also known as C_2_C_N_) and C_B_C_N_C_B_ (also known as C_2_C_B_), both in their neutral and ±1 charged states (if the charged state was stable); and pairs of nonadjacent C_B_–C_N_ defects known as donor–acceptor pairs (DAPs)^48^ with the required structural symmetry and pair distances below 7 Å. Following an initial screening based on DFT calculations,^49^ we kept the defects with the lowest spin-conserving single-particle transition between 1.5 and 3 eV (namely, a ±0.75 eV interval around the 2.25 eV experimental transition energy) and with a finite in-plane transition dipole. This left nine defects as the remaining candidates: C_N_V_B_^+^, C_N_N_B_, C_N_N_B_^+^, B_N_C_B_^–^, V_N_C_B_, V_N_C_B_^+^, C_B_V_N_C_B_^–^, and the two C_B_–C_N_ DAPs with pair distances *d* = 2.95 and 5.77 Å, respectively. Using the remaining criteria, further GW-BSE calculations ruled out all candidates except the C_B_–C_N_ DAP with a pair distance between carbon atoms of 5.77 Å, which is  times the lattice constant of hBN. Note that previously proposed carbon-based defects such as C_N_V_B_^–^ and C_2_C_N_^11,50,51^ have lower energy shelving states of the same spin multiplicity; the rate of internal conversion to such shelving states is on the order of MHz by theoretical estimates,^11,50,51^ which is 4 orders of magnitude larger than the *r*_23_ estimated for our type I emitter.

**Figure 5:**
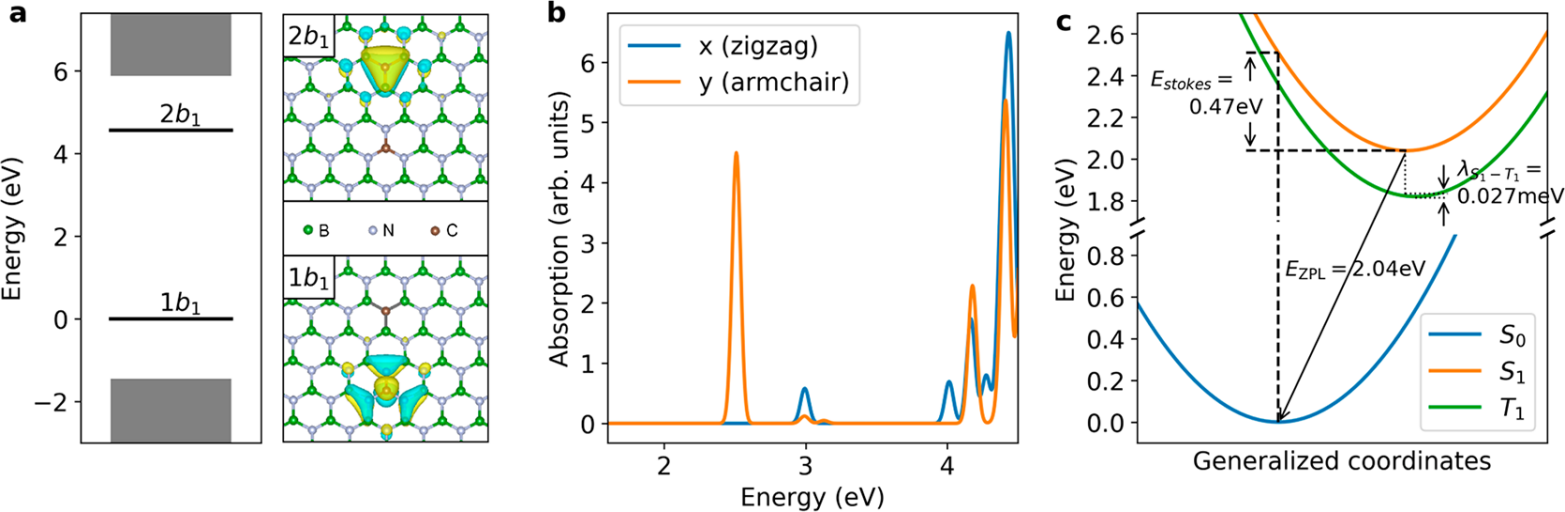
BSE calculations of the C_B_–C_N_ defect. (a) Left: energy levels of the C_B_–C_N_ donor–acceptor pair defect calculated with the GW method. The defect levels are denoted as 1b_1_ and 2b_1_ using the molecular orbital notation, and the bulk states are shown as shaded rectangles. Right: wave functions of the two defect states, shown together with the defect atomic structure. (b) BSE absorption spectra for two polarizations: *x*, parallel to the zigzag direction of the hBN crystal, and *y*, parallel to the armchair direction of the crystal. A small energy broadening of 0.02 eV was applied in the absorption spectra calculations. (c) Configuration energy diagram of the singlet ground state *S*_0_, the singlet excited state *S*_1_, and the triplet excited state *T*_1_ from constrained DFT and GW-BSE calculations. The difference between the equilibrium geometries of *S*_1_ and *T*_1_ and the corresponding reorganization energy λ_*S*_1_ – *T*_1__ are exaggerated in the plot.

The C_B_–C_N_ DAP defect proposed here has a planar *C*_2*v*_ structure with the C_B_ and C_N_ impurities separated by an hBN hexagonal unit cell. As shown in Figure 5a, this defect has a spin singlet ground state, and its single-particle energy levels consist of one occupied acceptor and one empty donor level inside the band gap, forming a clean two-level system. The wave functions of the two levels are localized around C_N_ and C_B_, respectively. In our GW-BSE calculations, the singlet exciton formed by the vertical transition between the donor and acceptor levels had an energy of 2.51 eV, a radiative lifetime of 16 ns,^49,52^ and a polarization along the direction of the pair. Additionally, as shown in Figure 5b, no other absorption peak exists within 0.5 eV of the first peak, consistent with the absence of higher energy peaks in our PLE measurements. To account for excited-state relaxation effects, we calculated the equilibrium geometry of the excited state using the constrained DFT approach by moving one electron from the acceptor level to the donor level in a spin-polarized DFT calculation. The resulting energy landscape, shown in Figure 5c, exhibits a Franck–Condon shift of 0.47 eV that leads to a ZPL energy of 2.04 eV, which is in good agreement with the 2.24 eV ZPL energy of the type I emitter. The predicted radiative lifetime of 16 ns is also consistent with the measured value of 4.4 ns.

The GW-BSE calculations also predicted a triplet exciton, formed by the same set of orbitals as the singlet, with an energy of 2.29 eV, which we tentatively assign to the shelving state. The radiative decay of the triplet state is spin-forbidden, explaining the small decay rate to the ground state (*r*_31_). Within the framework of the Franck–Condon principle, the rate of intersystem crossing from the emitter singlet state *S*_1_ to the shelving triplet state *T*_1_ can be described with Fermi’s golden rule:^53^
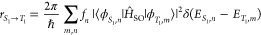
where *m* and *n* are the vibronic quantum numbers of the initial and final states, respectively, *f*_*n*_ is the thermal occupation factor, and *Ĥ*_SO_ is the spin–orbit coupling operator mixing the singlet and triplet states. Because these singlet and triplet states come from the same set of electronic orbitals, the first-order direct spin–orbit coupling between them is forbidden due to El-Sayed’s rule.^54^ Coupling is possible if the states are mixed by spin–orbit and vibronic coupling simultaneously, yet the strength of vibronic coupling is known to be subject to an exponential factor ,^53^ where Δ*E*_*S*_1_ – *T*_1__ is the energy splitting between the singlet and triplet states and λ_*S*_1_ – *T*_1__ is the energy change between the singlet and triplet equilibrium structures. Our constrained DFT calculation predicted λ = 0.027 meV, which is 4 orders of magnitude smaller than the singlet–triplet splitting Δ*E*_*S*_1_ – *T*_1__ = 0.22 eV, and thus, the vibronic coupling is strongly suppressed. This explains the small transition rate from the emitter to the shelving state (*r*_23_).

In conclusion, we identified and characterized a new type of single-photon emitter created by carbon implantation in hBN. These defect emitters exhibit more consistent and homogeneous properties than previously reported SPEs in hBN, such as a well-defined emission energy, dipole orientation, and excitation resonance. The predominant relaxation through a radiative channel marks their potential to be an efficient single-photon source for quantum optics experiments. Although post-tuning^55^ and stabilizing is still necessary, the substantially reduced energy inhomogeneity and limited polarization possibilities make overlapping energy and polarization between distinct emitters more achievable, which are needed for quantum interference. Based on these findings, future research can be directed at exploring improving wavelength tunability, looking into dephasing mechanisms^3,15,56^ and exploring experimental approaches to suppress spectral diffusion.^57^

## Methods

### Creation of the SPE Ensembles

The hexagonal boron nitride crystal was purchased from HQ Graphene (product number: BN2A1). It was mechanically exfoliated onto a silicon substrate with a 285 nm dry chlorinated thermal oxide layer, which was soaked in acetone and isopropanol for 5 min, respectively, to refresh the surface prior to exfoliation. After exfoliation, the wafer was brought to 12C+ implantation with 10 keV acceleration, a 0 tilt angle at 1 × 10^–6^ Torr under high vacuum at room temperature, and a dose of 1 × 10^13^ ions/cm^2^ (fulfilled by CuttingEdge Ions, LLC). Afterward, the wafer was annealed in 1 Torr of Ar gas at 900 °C for 30 min. We found the emitters in hBN flakes with varying thicknesses, from ∼10 nm to hundreds of nanometers. The specific sample we discussed in Figure 1a had a type I emitter density of 0.03 μm^–2^. Although the flake density varied, statistically the emitter density was higher in thicker flakes, which was likely caused by them receiving more carbon ions during the implantation process.

### Photoluminescence

The raster scan for the identification of emitters was conducted at room temperature. For excitation, we used a 532 nm laser that was circularly polarized such that emitters with any polarization direction were unbiasedly detected. A high numerical aperture (NA = 0.95) objective lens was used to effectively collect photoluminescence from the sample. It was mounted on a nanopositioner with subnanometer piezo-based position control, which allowed us to scan across the sample during which one spectrum was taken at each pixel. Then, the spectra were analyzed to identify the positions of the emitters. For the polarization-resolved measurements in Figure 1e,f, the same 532 nm laser was used; we placed a linear polarizer in both the excitation and detection paths, used a half waveplate for each to adjust the excitation laser linear polarization, and detected the photoluminescence linear polarization.

### Photoluminescence Excitation (PLE)

A schematic of the PLE setup is shown in Figure S3. The wavelength selection was realized by sending a supercontinuum laser (20 MHz rep rate, 6 ps pulsewidth) through an optical setup with a 4f system terminated by a grating on both sides in symmetrical configuration. The first grating was responsible for spectrally dispersing the beam. After the desired wavelength was selected by a slit, a second grating diverted the selected beam toward the same direction regardless of the selected wavelength. This configuration allowed us to select the wavelength by only moving the slit by a translation stage without adjusting the rest of the setup. The output beam for excitation was then tuned into circular polarization such that no linear polarization direction was favored while we explored the states that might exhibit any dipole orientation.

### Photon Autocorrelation

The emitters were excited with a 532 nm continuous laser. The laser polarization was aligned to the emitter’s absorption polarization for efficient excitation. In the detection path, we used a 532 nm notch filter to block the excitation laser while we sent the photoluminescence signal to a 50/50 beam splitter. At the end of each arm, there was a single-photon counter (SPCM-cd-3017) for collection. The photon incidences were time-tagged by a PicoQuant TimeHarp 260 for analysis. To take into account the background luminescence from hBN, the experimental *g*_exp_^2^ was then background-corrected by
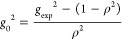
where ρ = SNR/(SNR – 1) and SNR is the signal-to-noise ratio.

### Theoretical Calculations

We carried out spin-polarized DFT calculations on atomic defects in hBN with the Quantum ESPRESSO package.^58,59^ We used the Perdew–Burke–Ernzerhof (PBE) exchange-correlation functional on 6 × 6 × 1 supercells of monolayer hBN containing one defect. The GW-BSE calculations were performed with the YAMBO code^60,61^ using a 2 × 2 × 1 *k*-point grid for the dielectric function. Our previous study showed that the parameters used in these calculations can converge the exciton energy within 0.1 eV.^49^ Exciton radiative lifetimes were computed from the exciton wave function using Fermi’s golden rule and taking into account the dielectric screening of the crystal environment.^49,52^ The DFT and GW-BSE calculation results for additional defect candidates are available in the Supporting Information.

## Supporting Information Available

Additional details and figures for the implantation ion control experiment, dipole orientation data for the type II emitters, PLE setup schematics, more type I and type II PLE results, power-dependent autocorrelation details, and computed properties of carbon-related defects (PDF)
