# Supplementary material for: Carbon-Related Quantum Emitter in Hexagonal Boron Nitride with Homogeneous Energy and 3-Fold Polarization
Source: Nano Lett. 2024 Jan 19;24(4):1106–13. doi: 10.1021/acs.nanolett.3c03628 (PMC10835729; doi:10.1021/acs.nanolett.3c03628)
Supplement: Supplementary file 1 — nl3c03628_si_001.pdf [file nl3c03628_si_001.pdf]

Supporting Information for

Carbon-Related Quantum Emitter in Hexagonal Boron Nitride with Homogeneous Energy and Three-fold Polarization

Authors: Ding Zhong[1,2,3], Shiyuan Gao[4], Max Saccone[5], Julia R. Greer[6], Marco Bernardi[4], Stevan Nadj-Perge[1,3], Andrei Faraon[1,2,3*]

Affiliations:
[1]Thomas J. Watson, Sr., Laboratory of Applied Physics, California Institute of Technology, Pasadena, CA 91125, USA.
[2]Kavli Nanoscience Institute, California Institute of Technology, Pasadena, CA 91125, USA.
[3]Institute for Quantum Information and Matter, California Institute of Technology, Pasadena, CA 91125, USA.
[4]Department of Applied Physics and Material Science, California Institute of Technology, Pasadena, CA 91125, USA.
[5]Division of Chemistry and Chemical Engineering, California Institute of Technology, Pasadena, CA 91125, USA
[6]Division of Engineering and Applied Science, California Institute of Technology, Pasadena, CA 91125, USA

[*]Correspondence to: faraon@caltech.edu

Contents:
S1. The Fabrication Control: O Implantation.
S2. Absorption/emission dipole orientation for type II emitters.
S3. Schematics for PLE setup
S4. Additional PLE results of type I and type II emitters.
S5. Excitation power-dependent autocorrelation.
S6. Computed Properties of Carbon Related Defects.

S1. The Fabrication Control: O Implantation.

The creation of type I emitters using carbon implantation suggests carbon related defect origin. However, this evidence on its own is not enough to rule out possibilities such as creation of intrinsic defects during implantation. Thus, we conducted a control experiment in which $^{16}O^+$ ions are implanted, with the implantation parameters remaining the same.

Figure S1.a and b shows the raster scan results before and after the $^{12}C^+$ (10 keV, 0° angle, room temperature) and annealing (900 °C, 1 Torr Ar, 30 min), with each pixel's color denoting the average intensity between [1.8, 2.25] eV, which include both the type I and type II energy. There is an increased number of the emission hotspots in the region, owning to emerging of new emitters after the implantation. We further plot the same plot with the color representing the average

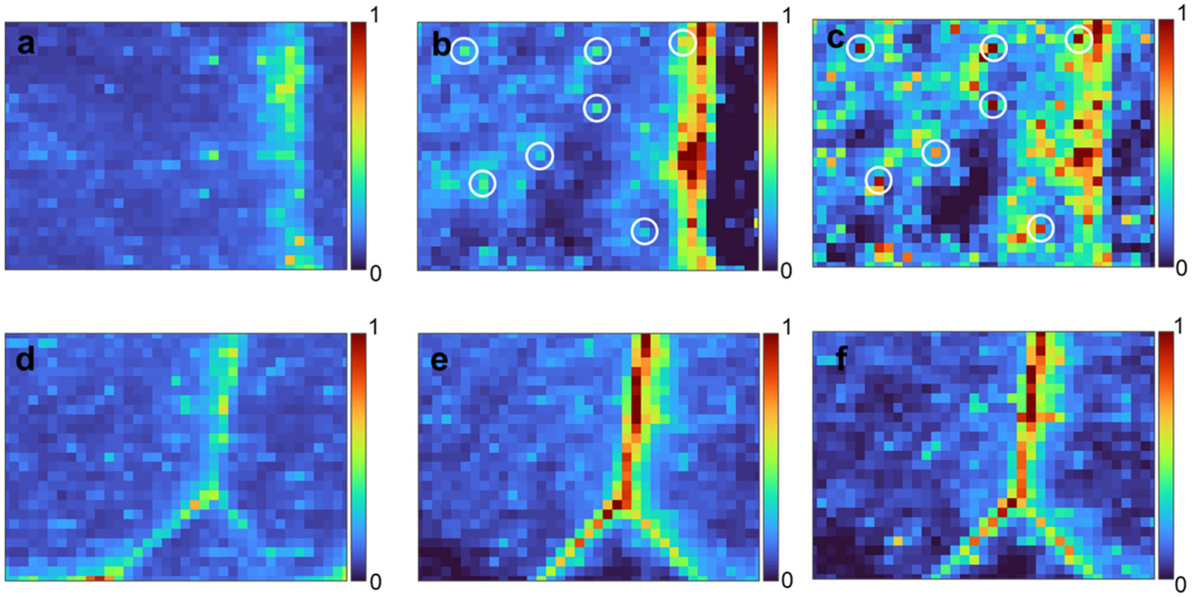

Figure S1. Controlling dopant for creating type I emitters. (a) PL raster scan of a portion of the flake after mechanical exfoliation, prior to any treatment. Color represents average intensity between [1.8, 2.25] eV. (b) The scan over the same area after $^{12}C^+$ implantation and annealing. Circles mark locations of confirmed type I emitters. (c) The same image as in (b) but plotting the average intensity between [2.19 2.25] eV, at the type I emitter's energy. (d) Raster scan of another flake prior to implantation with $^{16}O^+$. (e) The scan over the same area after $^{16}O^+$ implantation and annealing. (f) The same image of (e) but showing the average intensity between [2.19 2.25] eV, at the type I emitter's energy. No type I emitter is found.

intensity between [2.19, 2.25] eV, centered around only the type I emitter. The majority of the hotspots are preserved in Figure S1c, indicating that these are type I emitters, as circled out by the white marker. This result is verified by individually examining the spectrum at such spots.

The same types of figures are plotted for the $^{16}O^+$ implanted sample in Figure 1 d-f. There are minor changes in the number and locations of emission hotspots in Figure S1e compared to Figure S1d. These are due to activation and migration of defects during implantation and annealing. There are no strong hotspots in Figure S1f, which only capture intensity around type I emitters' energy. As we examine the spectrum pixel by pixels, we did not find any type I emitter, and some of the relatively high signal spots are from broad background signal.

The control experiment negates the possibility that the type I emitter is an intrinsic defect. If it were intrinsic, the $^{16}O^+$ implantation would be able to displace intrinsic atoms to new sites, potentially forming the same intrinsic emitters, which is not evidenced by our observation. The slight difference in mass should not entirely prevent the formation of the same intrinsic defects. Additionally, the experimental data negates the possibility of emitter association with oxygen atoms, which may have been inadvertently introduced during hBN growth or through hydrocarbon contamination on the surface.

As a side note, we would like to note the density of type I emitters on each flake has variations. There is no reliable indicator found to predict the density of emitters on each flake, although in general we locate more type I emitters on thicker flakes. This is likely attributed to thicker flakes having larger effective collision cross section that intercepts more ions from the beam.

S2. Absorption/emission dipole orientation for energy-preselected type II emitters.

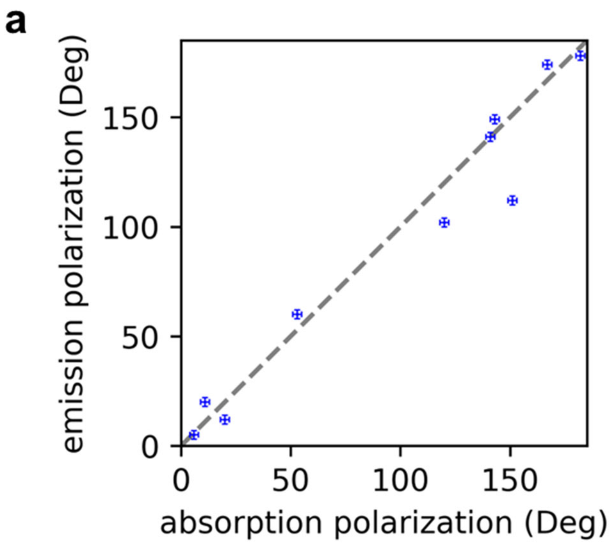

Figure S2. Absorption/emission dipole orientation for 10 type II emitters between 580 and 590 nm. (a) The scatter plot of 10 type II emitters with close emission energy, with the error bar shown. The gray line serves a guide for eye as absorption dipole angle align with emission dipole angle. This data reveals no discernible pattern in the absorption or emission dipole orientations.

S3. Schematics for PLE setup.

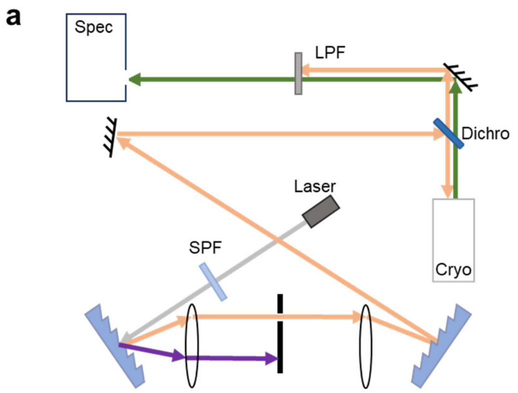

Figure S3. Schematics of PLE setup. (a) The laser from the supercontinuum is first being cut off by a short pass filter (SPF). Then a grating disperses the light, which passes through a slit on a translational stage, leaving a semi-monochromic beam. The secondary grating in conjugate position disperses the beam back to a position regardless of selected wavelength. A dichroic mirror reflects the excitation light towards to cryostat (Cryo). The single photon emission together with the remnant of excitation light goes through a long pass filter (LPF) where the latter is filtered. The spectrometer (Spec) records the spectrum. Polarization components are added depending on experiment needs (not shown).

S4. Additional PLE results of type I and type II emitters.

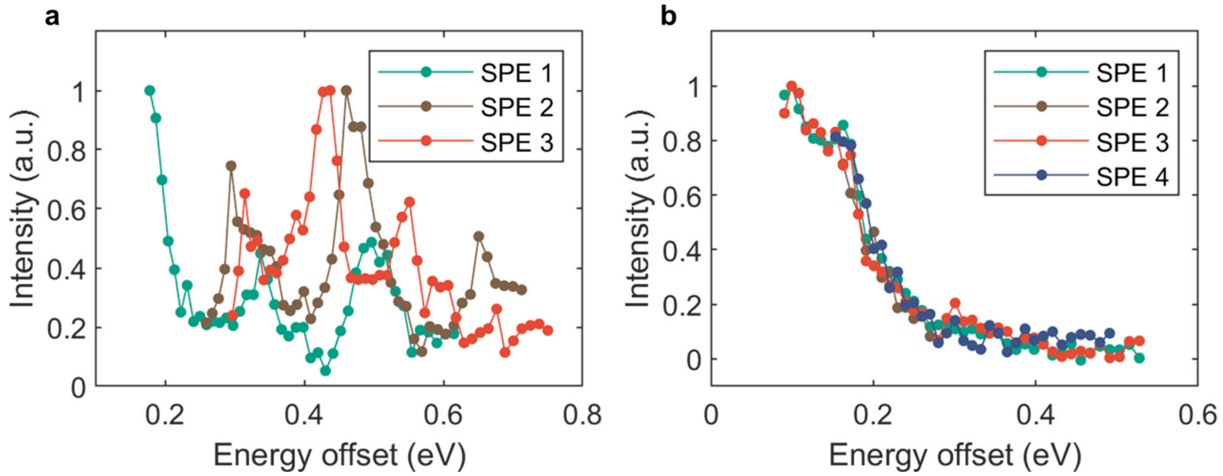

Figure S4. Additional PLE results. (a) Three more type II emitters results are presented. They are plotted versus energy offset from their respective emission energies: 2.15 eV for SPE 1, 2.08 eV for SPE 2 and 2.04 eV for SPE 3. No consistent patterns are observed among these results. (b) Four type I emitters PLE results are presented, exhibiting highly reproducible PLE patterns.

S5. Excitation power-dependent autocorrelation.

Assuming a power dependent excitation rate $r_{12}=\beta * P$, we could obtain these coefficients quantitatively by conducting the experiment with different power. First of all, the decay rate $\lambda_1 = r_{12} + r_{21}$ approaches asymptotically to r_{21} in the zero-power limit, allowing for obtaining r_{21} by

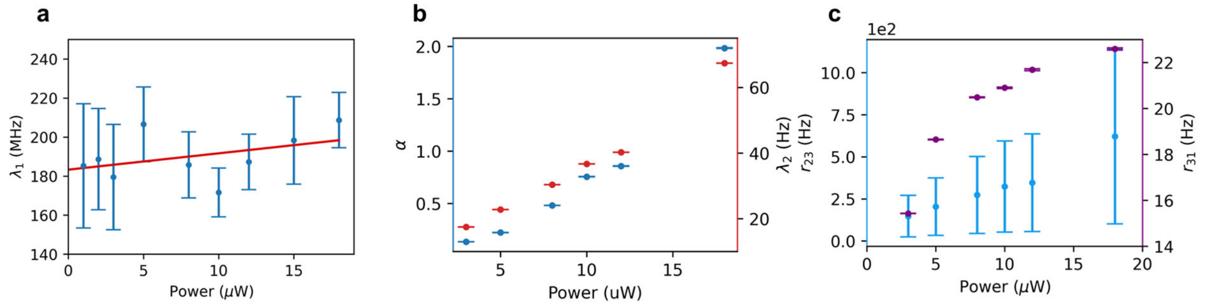

Figure S5. Excitation Power dependent parameters. a, Antibunching decay rate λ_1 as function of power. The red line is a linear fit with y-axis intercept at 183.3 MHz. **b**, Dependence of the bunching amplitude α and decay rate λ_2 on the excitation power. **c**, Transition coefficients r_{23} and r_{31} as function of power.

extrapolation. As shown in Figure S5a, we obtained a relaxation rate of $r_{21} = 183.3$ MHz, which corresponds to a lifetime $t_1 = 5.5$ ns. In addition, r_{23} and r_{31} can be extracted from the power dependence of λ_2 and α, as shown in Figure S5b, c. We find that r_{31} increases rapidly and levels off at high power, which can be explained by a faster excitation rate depopulating the ground state, leading to a faster deshelving rate from the metastable state. The same power dependence is also observed for single molecules[1] and color centers in diamond[2]. r_{23} is only several hundred Hertz, more than 5 orders of magnitude smaller than r_{21}. In fact, as seen from the expression of λ_2 above, low transition rates r_{23} and r_{31} can be inferred from a small λ_2, which manifest as long bunching characteristic decay time. In single photon generation scenario, where the emitter is excited by a laser pulse, the extremely low r_{21}/r_{23} ratio leads to a dominant relaxation through radiative channel, making it a highly efficient single photon source.

S6. Computed Properties of Carbon-Related Defects

A computational screening is done for carbon-related defect candidates that satisfy the structural symmetry constrain, as discussed in the main text. Table S1 shows the results of the calculations. In this table, results of DFT and constrained DFT calculations are shown for all the defect candidates, while GW+BSE calculations are carried out for most of the defects except those with a single-particle transition energy far away from 2 eV. Notably, the defects C_NN_B and $C_BV_NC_B^-$ (negatively charged $C_BV_NC_B$) also have exciton energy close to the observed emitter. However, they are not preferred over the d=5.77 Å C_B-C_N DAP because (1) they have very low brightness with much higher radiative lifetimes than the experimental value; (2) for C_NN_B, higher absorption peaks near the first peak is also present, which is inconsistent with the PLE spectrum for type I emitter.

type	defect symbol	ground state spin polarization	single-particle transition (PBE, eV)	ZPL (constrained DFT, eV)	singlet exciton (BSE, eV)	radiative lifetime (BSE, ns)
double-site defects	C_NV_B	1	1.15	1.05-0.11	1.02	8.0×10^4
	$C_NV_B^-$	1/2	0.44	0.72-0.42		
	C_NN_B	1/2	2.20	2.53-0.18	2.36	4500
	$C_NN_B^+$	0	2.44	2.38-0.20	3.47	14
	B_NC_B	1/2	1.10	1.11-0.37		

	$B_NC_B^-$	0	1.70	2.15-0.20	1.27	8500
	V_NC_B	0	1.93	1.82-0.78	2.61	121
	$V_NC_B^+$	1/2	1.70	1.69-0.90	1.58	640
	$V_NC_B^-$	1/2	0.42	0.62-0.39		
triplet-site defects	$C_NV_BC_N$	1/2	1.49	1.34-0.18	0.78*	9400
	$C_NV_BC_N^-$	0	0.23	0.50-0.37		
	$C_BV_NC_B$	1/2	1.28	1.24-0.29		
	$C_BV_NC_B^-$	0	2.07	1.98-0.09	2.47*	1.4×10^4
	$C_NC_BC_N$	1/2	1.12	1.27-0.12		
	$C_BC_NC_B$	1/2	1.23	1.29-0.19		
donor-acceptor pairs (DAPs)	C_B-C_N d=2.95 Å	0	2.37	2.76-0.33	3.46	4.4
	C_B-C_N d=5.77 Å	0	1.39	2.27-0.47	2.51	16
	C_B-C_N d=7.21 Å	0	1.10	2.16-0.50		

Table S1. Calculated properties of carbon-related defects. The superscripts "+" and "-" in the defect symbol column indicates +1 and -1 charge state of the defect, respectively. The single-particle transition energy is the energy difference between the lowest unoccupied and highest occupied band of the same spin species within PBE. The ZPL energy is written as the vertical excitation energy minus the Stokes shift, calculated within PBE by forcing the occupation of the highest occupied band (HOB) to be 0 and lowest unoccupied band (LUB) to be 1. Typically, the HOB-LUB transition is the main component of the exciton wave function, with a few exceptions indicated by an asterisk (*) on the exciton energy column.

References:

1. Treussart, F., Clouqueur, A., Grossman, C. & Roch, J.-F. Photon antibunching in the fluorescence of a single dye molecule embedded in a thin polymer film. *Opt. Lett.* **26**, 1504–1506 (2001).
2. Wu, E. *et al.* Narrow-band single-photon emission in the near infrared for quantum key distribution. *Opt. Express* **14**, 1296 (2006).
